# Markers of adiposity among children and adolescents: implications of the isotemporal substitution paradigm with sedentary behavior and physical activity patterns

**DOI:** 10.1186/s40200-015-0175-9

**Published:** 2015-05-27

**Authors:** Paul D. Loprinzi, Bradley J. Cardinal, Hyo Lee, Catrine Tudor-Locke

**Affiliations:** Department of Health, Exercise Science, and Recreation Management, Center for Health Behavior Research, School of Applied Sciences, The University of Mississippi, 215 Turner Center, University, MS USA; Program in Exercise and Sport Science, School of Biological and Population Health Sciences, College of Public Health and Human Sciences, Oregon State University, Corvallis, OR USA; Department of Sport and Health Sciences, Sangmyung University, Seoul, South Korea; Walking Behavior Laboratory, Population and Public Health Sciences, Pennington Biomedical Research Center, Baton Rouge, LA USA

**Keywords:** Accelerometry, Epidemiology, Isotemporal substitution models, NHANES, Partition models

## Abstract

**Background:**

The purpose of this study was to examine the association between daily movement patterns and dual energy X-ray absorptiometry-determined body fat percent (DXA-BF%) among children and adolescents while applying both traditional and novel analytical procedures.

**Methods:**

Using data from the cross-sectional 2003–2006 National Health and Nutrition Examination Survey (*n* = 5607), physical activity was assessed via accelerometry, with the following movement patterns assessed: 1) meeting moderate-to-vigorous physical activity (MVPA) guidelines and engaging in more light-intensity physical activity (LIPA) than sedentary behavior (SB); 2) meeting MVPA guidelines, but engaging in less LIPA than SB; 3) not meeting MVPA guidelines, but engaging in more LIPA than SB; and 4) not meeting MVPA guidelines and engaging in less LIPA than SB. Various markers of adiposity (e.g., DXA-BF%) were assessed.

**Results:**

Children in movement pattern 1 (52 %), compared to those in movement pattern 4, had significantly lower levels of BMI (∆ 2.2 kg/m^2^), waist circumference (∆ 6.5 cm), tricep skinfold (∆ 4.2 mm), subscapularis skinfold (∆ 2.6 mm), android BF% (∆ 7.6 %), gynoid BF% (∆ 5.1 %), and total BF% (∆ 5.2 %). Substituting 60 min/day of SB with MVPA resulted in a 4.6 % decreased estimate of total DXA-BF%. No findings were significant for adolescents.

**Conclusions:**

The low proportion of children engaging in ≥ 60 min/day of MVPA and accumulating relatively more LIPA than SB had the lowest DXA-BF%.

## Introduction

Accumulating evidence depicts an inverse association between objectively-measured moderate-to-vigorous physical activity (MVPA) and adiposity markers among children and adolescents [1]. However, the majority of these studies have exclusively relied upon proxy markers of adiposity (e.g., body mass index; BMI) [1] and the potentially latent contributory influence of lighter intensity activities has been largely ignored. Replacing sedentary behaviors (SB; i.e., those that produce little to no movement) with increased amounts of light-intensity physical activity (LIPA; i.e., physical activities performed at intensities less than MVPA) represents an overlooked adjunct strategy for mitigating excessive adiposity in this younger population [2, 3]. Additional research examining these understudied behaviors, separate and combined with MVPA, is warranted, as there is mixed evidence regarding their potential beneficial effects [2, 4–6].

In addition to using traditional analytical models to study the independent effects of SB, LIPA, and MVPA on select health outcomes such as adiposity [7], recent work has employed novel approaches. For example, Loprinzi, Lee, and Cardinal [8], as well as others [9], have provided prevalence estimates of movement patterns and their effects on health outcomes. These movement patterns consist of 4 distinct ascribed categories, including: 1) participants meeting MVPA guidelines and engaging in relatively more LIPA than SB (i.e., the ratio LIPA: SB ≥ 1); 2) meeting MVPA guidelines and engaging in relatively less LIPA than SB; 3) not meeting MVPA guidelines, but engaging in relatively more LIPA than SB; and 4) not meeting MVPA guidelines and engaging in relatively less LIPA than SB. Hereafter we refer to these classifications as *movement patterns* by numbers 1–4, with 1 and 4, respectively, considered the most and least active individuals. Similarly, hereafter the term *‘behavior’* will be used to describe SB, LIPA, and MVPA.

The prevalence of these movement patterns and their associations with markers of adiposity have only been reported for Belgian children and adults [9] or U.S. adults [8] thus far. To our knowledge, no one has studied these movement patterns yet in U.S. children and adolescents. Additional analytical innovation [10–13] has employed isotemporal substitution models to examine the substitution effect of replacing one behavior for another for an equal amount of time on a select outcome of interest (e.g., the effect of replacing 60 min/day of SB with 60 min/day of MVPA on body fat percent [BF%]). However, to our knowledge, this approach has not been applied to data representing U.S. children and adolescents.

The purpose of this secondary analysis of the 2003–2006 National Heatlh and Nutrition Examination (NHANES) accelerometer data was to use these traditional and more novel analytical approaches to systematically examine the association of SB, LIPA, and MVPA behaviors and movement patterns with markers of U.S. children’s and adolescents’ objectively-measured whole-body dual energy X-ray absorptiometry BF (DXA-BF%), in addition to other proxy markers of adiposity (e.g., BMI, waist circumference and skinfold thickness). More specifically, here we examine the independent and combined associations of behaviors and movement patterns on several markers of adiposity among U.S. children and adolescents.

## Methods

### Design

Data from the present study were extracted from the cross-sectional 2003–2006 NHANES, an ongoing survey conducted by the Centers for Disease Control and Prevention that uses a representative sample of non-institutionalized U.S. civilians, selected by a complex, multistage, stratified, clustered probability design. Participants are interviewed in their home and subsequently examined in a mobile examination center (MEC). While at the MEC, participants complete surveys, anthropometric measurements (e.g., waist circumference and dual energy X-ray absorptiometry scanning), and participants are then given an accelerometer to wear for the subsequent 7 days. For further assessment of certain parameters (e.g., diet), participants are contacted for a follow-up telephone interview. Additional details about NHANES protocols have been published (http://www.cdc.gov/nchs/nhanes.htm).

The 2003–2006 NHANES study procedures were approved by the National Center for Health Statistics ethics review board. Assent was obtained from all participants prior to any data collection.

### Demographics/Covariates

Covariates included age, gender, race-ethnicity (Mexican American, non-Hispanic white, non-Hispanic black, and other race), energy intake, poverty-to-income ratio (PIR), cotinine, and accelerometer wear time. Age, gender, and race-ethnicity were obtained from a questionnaire. Energy intake (kcal) was assessed from the MEC interview and the follow-up telephone interview, with the average of these values used; if data was missing from the telephone interview, only energy intake obtained from the MEC interview was used. Parent-proxy interviews were conducted for children under 9 years, with children 9–11 years permitted to receive assistance from their parent.

As a measure of socioeconomic status, PIR was assessed (a value < 1 was considered below the poverty threshold) [14, 15]. The PIR is calculated by dividing the family income by the poverty guidelines, which is specific to the family size, year assessed, and state of residence. Serum cotinine was measured by an isotope dilution-high performance liquid chromatography/atmospheric pressure chemical ionization tandem mass spectrometry.

### Markers of adiposity

Markers of adiposity included height, weight, waist circumference, tricep and subscapularis skinfold, gender-specific BMI-for-age percentile [16], android-specific BF%, gynoid-specific BF%, and total BF%. BF% was estimated from whole-body dual energy X-ray absorptiometry scans using the Hologic QDR 4500A fan beam x-ray bone densitometer (Hologic, Inc, Bedford, Massachusetts).

### Accelerometry

Participants were asked to wear an ActiGraph 7164 accelerometer for 7 days, except while engaging in water-based activities and while sleeping. Detailed information on the ActiGraph accelerometer and the protocol implemented in NHANES can be found elsewhere [17, 18].

### Data treatment

#### Analytic sample

A total of 5607 children (6–11 years) and adolescents (12–17 years) were enrolled in the 2003–2006 NHANES cycles. The analytic sample comprised 2856 children and adolescents after excluding those with missing covariate data, specifically age, gender, race-ethnicity, cotinine, energy intake (kcal), PIR, and those with insufficient (<4 days of 10+ hrs/day of monitoring) or missing accelerometry data. There were no differences across gender (*p* = 0.31) between those excluded based on missing/insufficient accelerometry data and the analytic sample. However, those who were excluded were younger (10.1 vs. 12.1 years vs., *p* < 0.01), had a smaller BMI (20.8 vs. 21.3 kg/m^2^, *p* < 0.01), had a higher cotinine level (7.5 vs. 4.7 ng/mL, *p* < 0.01), had a lower PIR (1.9 vs. 2.1, *p* < 0.01), and were more likely to be non-Hispanic white (28.5 vs. 25.3 %, *p* < 0.01). These estimates are unweighted.

#### Accelerometry

SAS (version 9.2) was used to reduce accelerometry data to those with ≥ 4 days of ≥ 10 h/day of monitored data [19] and integrate it into 1 min time intervals using the SAS syntax provided by the National Cancer Institute (NCI). Nonwear time was identified as ≥ 60 consecutive minutes of zero activity counts, with allowance for 1–2 min of activity counts between 0 and 100 [19]. Accelerometer wear time (in hours) was calculated by subtracting nonwear time from 24 h. The Freedson age-specific cut-points imbedded in the NCI SAS syntax were used to determine time spent in moderate physical activity (MPA) and vigorous physical activity (VPA) [20]. We also explored associations using Evenson [21] non-age specific MVPA cut-point of 2296 cpm; however, these results were similar to the Freedson age-specific MVPA cut-points so the Freedson cut-points were utilized in the present study. Indeed, previous work has shown very similar classification accuracy between the Evenson and Freedson age-specific cut-points among children and adolescents [22]. The NCI SAS syntax was edited to classify SB as accumulated time with activity counts/min ≤ 99 [23], and LIPA as activity counts/min ≥ 100 and below the age-specific MPA cut-point. Total physical activity (TPA) was defined as LIPA + MVPA, or anything not classified as SB. Accelerometry data were also used to identify the four mutually exclusive movement patterns described in the introduction section [8, 9, 24]. Ratios of MVPA to SB (MVPA:SB), LIPA to SB (LIPA:SB), and TPA to SB (TPA:SB) greater than or equal to 1 indicate that participants engaged in relatively more MVPA, LIPA, or TPA than SB. Determination of meeting MVPA guidelines was rendered with evidence of accumulating ≥ 60 min/day of MVPA [25].

#### BMI

BMI was calculated as weight in kilograms divided by the square of height in meters.

### Statistical analysis

#### Sample weights

All statistical analyses were performed using procedures from sample survey data using Stata (version 12.0, College Station, TX). The MEC sample weights were used to account for oversampling, non-response, non-coverage, and to provide nationally representative estimates. In an effort to maintain nationally representative estimates, the sample weights for the reduced sample with ≥ 4 valid accelerometry data were ratio-adjusted to maintain the age, gender, and race-ethnicity distribution of the full sample. These sampling weights were then recalcuated to account for the combination of both NHANES cycles (i.e., 2003–2004 and 2005–2006).

#### Statistical significance

Statistical signifiance was established as a Bonferroni-corrected *p* < 0.006 (8 different markers of adiposity evaluated; alpha of 0.05/8 = 0.006).

#### Behavior estimates across age

To assess differences in behavior estimates across the age-groups (children vs. adolescents), adjusted Wald tests and design-based likelihod ratio tests, respectively, were used for continuous and categorical variables.

#### Movement patterns

Multivariable linear regression analysis was used to examine the association between the movement patterns and each marker of adiposity (outcome variable). Separate models were examined for each marker. The referent group was movement pattern 4 (considered the least active combination of behaviors).

#### Single behavior, partition and isotemporal substitution models

Three additional types of regression models were fitted that included single behavior models, a partition model, and isotemporal substitution models; detailed explanations of these models can be found elsewhere [10, 12]. Prior to implementing these regression models, each behavior was divided by a constant of 60 so that a unit increase in each consistently represented an increase of 60 min/day for that behavior.

#### Single behavior

The purpose of the single behavior models were to examine the total effect for each behavior [10]. Three separate models were computed: a regression model examining the association between SB and adiposity, with additional models for LIPA and MVPA.

#### Partition model

The purpose of the partition model was to examine the unique effect for each behavior [10]. In this partition model, all three behaviors were entered into the model at the same time. All previously listed covariates were entered into this model with exception of accelerometer wear time (hrs/day). This exception was necessary because accelerometer wear time is equal to the sum of SB, LIPA, and MVPA behaviors. Importantly, there was no evidence of multicollinearity in the partition model or other models; evidence of multicollinearity is likely to be present if the pairwise correlation between two variables is > 0.8 (highest observed, *r* = 0.59), if the mean variance inflation factor is > 6 (observed mean = 1.3), if the highest individual variance inflation factor is > 10 (highest observed = 2.1); or if the tolerance statistic is < 0.1 (all observed to be > 0.47).

#### Isotemporal substitution model

The purpose of the isotemporal substitution models was to estimate the substitution effect of replacing one behavior for a different behavior [10]. This was accomplished by entering a total combined behavior variable (time spent in SB + LIPA + MVPA, which is also equal to accelerometer wear time) along with each specific behavior variable into the model at the same time. The specific behavior variable of interest is then dropped from the model. Using this systematic analytical approach, isotemporal substitution models can demonstrate the effect that replacing one behavior for another has on adiposity (e.g., substituting 60 min/day of SB with MVPA results in a given change in BF%) [10, 12]. Similar to the partition model, accelerometer wear time (hrs/day) was not included as a covariate in the isotemporal substitution model. Also, it is not possible to examine the effects of replacing SB with TPA because for isotemporal substitution analysis you have to keep TPA in the model and then drop the variable of interest, which in this case would be TPA.

#### Multiple imputation for dual energy x-ray absorptiometry-BF%

Children below 8 years of age were not eligible for the dual energy X-ray absorptiometry scans; therefore, BF% estimates reported herein are for children and adolescents 8–17 years. Examination of the dual energy X-ray absorptiometry data showed that missing data for total BF% demonstrated a systematic, non-random pattern; therefore, only assessing participants with measured data for total BF% would lead to biased results. Therefore, missing dual energy X-ray absorptiometry values for total BF% were imputed using multiple imputation procedures (i.e., sequential regression multivariate imputation) [26, 27], ultimately generating 5 total BF% values for each participant.

#### Calculating imputed DXA-determined total BF% and variance estimate

Detailed information on providing estimates for the multiple imputated dual energy X-ray absorptiometry data can be found elsewhere [26, 28]. Briefly, estimation procedures were applied to each of the 5 versions of the imputed data. For the total BF% data, we calculated the BF% estimate (either the mean or the regression coefficient) and its associated standard error for each of the imputed values, resulting in 5 different estimates. The combined total BF% estimate was calculated as the mean of the 5 individual estimates. However, the combined standard error for this estimate is based on the calculation of the within-imputation variance (W) and the between-imputation variance (B). The W is the mean of the 5 individual variance estimates, and the B is the sample variance which is calculated as:

$$ \mathrm{B}={{\displaystyle \sum \left(\mathrm{Qi}\hbox{-} \mathrm{Q}\right)}}^2/4 $$

where the Qi’s are the individual variance estimates and Q is the mean of the 5 individual estimates.

The total variance (T) was then calculated as:

$$ \mathrm{T}=\mathrm{W}+\left(6/5\right)*\mathrm{B} $$

The square root of T was calculated as this represents the combined standard error associated with the combined estimate of Q.

#### Determining statistical significance from imputed estimates

This procedure (i.e., calculation of Q and square root of T) was performed for each total BF% regression coefficient. This ‘adjusted’ regression coefficent and ‘adjusted’ variance estimate were divided (i.e., β / square root of T) to produce the corresponding regression *t*-value. To determine whether this ‘adjusted’ *t*-value was statistically significant, we used 30 as the degrees of freedom (the number of primary sampling units minus the number of sampling strata). If the ‘adjusted’ *t*-value was greater than the *t*-value associated with 30° of freedom and a Bonferroni-corrected alpha of 0.006 (i.e., a two-tailed *t*-value of ± 2.95), then the regression coefficient was considered statistically significant.

## Results

### Physical activity and SB estimates across age-group

Table 1 shows the weighted behavior estimates for children and adolescents. Children wore the accelerometer less than adolescents (13.6 vs. 14.2 h/day) (*p* < 0.001). Children engaged in more LIPA, MPA, VPA, MVPA, and TPA, and engaged in less SB than adolescents (*p* < 0.001 for all). For children, the weighted proportions for movement patterns 1, 2, 3, and 4, respectively, were 52.2, 18.8, 10.1 and 18.7 %. For adolescents, the respective weighted proportions were 4.7, 5.2, 17.0 and 72.9 %.

Results were similar between genders (not shown in tabular format). For children, the weighted proportions across the movement patterns for boys were 55.1, 23.1, 5.8 and 15.7 %. For female children, the weighted proportions were 49.1, 14.0, 14.8 and 22.0 %.

For adolescents, the weighted proportions across the movement patterns for boys were 8.2, 8.7, 20.1 and 62.8 %. For female adolescents, the weighted proportions were 1.0, 1.4, 13.8 and 83.6 %. Given the small sample size for movement patterns 1 and 2 separately for males and females, associations with markers of adiposity were not stratified by gender.

**Table 1:** Weighted mean/proportion (95 % CI) sedentary and physical activity estimates across age group, NHANES 2003–2006

Accelerometer-Determined Behavior	Children (6–11 years) (*n* = 1036)	Adolescents (12–17 years) (*n* = 1608)	*P*-Value^a^
SB (min/day)	351.7 (345.0–358.4)	480.6 (470.2–491.1)	<0.001
LIPA (min/day)	382.5 (377.1–387.9)	343.0 (335.5–350.5)	<0.001
MPA (min/day)	73.7 (70.9–76.5)	24.9 (22.8–27.1)	<0.001
VPA (min/day)	13.2 (12.2–14.2)	3.6 (3.1–4.1)	<0.001
MVPA (min/day)	86.9 (83.5–90.3)	28.6 (26.0–31.2)	<0.001
TPA (min/day)	469.4 (462.4–476.5)	371.6 (363.4–379.9)	<0.001
% ≥ 60 min/day of MVPA	71.0 (66.7–75.4)	9.9 (7.5–12.4)	<0.001
Accelerometer wear time (hr/day)	13.6 (13.5–13.8)	14.2 (14.0–14.4)	<0.001
Movement Patterns, %			<0.001
≥60 min/day MVPA and LIPA:SB ≥ 1	52.2 (47.3–57.2)	4.7 (3.2–6.3)	
≥60 min/day MVPA and LIPA:SB < 1	18.8 (15.5–22.0)	5.2 (3.7–6.6)	
<60 min/day MVPA but LIPA:SB ≥ 1	10.1 (7.3–13.0)	17.0 (13.8–20.2)	
<60 min/day MVPA and LIPA:SB < 1	18.7 (16.0–21.3)	72.9 (69.8–76.0)	

*SB* sedentary behavior, *LIPA* light-intensity physical activity, *MPA* moderate physical activity, *VPA* vigorous physical activityi, *MVPA* moderate-to-vigorous physical activity, *TPA* total physical activity (LIPA + MVPA)

^a^Adjusted Wald test used to test for differences across continuous variables. Design-based likelihood ratio test used to test for differences across categorical variables (e.g., whether they engaged in ≥ or < 60 min/day of MVPA)

**Fig. 1:**
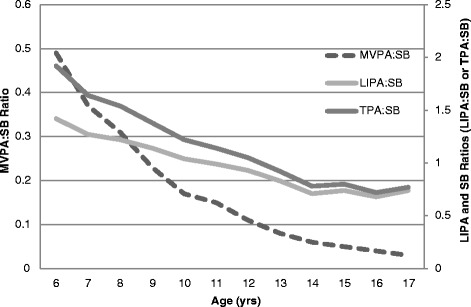
Weighted MVPA:SB, LIPA:SB and TPA:SB estimates with age (MVPA:SB = moderate-to vigorous physical activity to sedentary behavior ratio; LIPA:SB = light-intensity physical activity to sedentary behavior ratio; TPA:SB = total physical activity (LIPA + MVPA) to sedentary behavior ratio). A ratio > 1 indicates the participant engaged in more relative physical activity than SB

Figure 1 shows the *cross-sectional* associations of MVPA:SB, LIPA:SB, and TPA:SB with age. With progressive *cross-sectional* increments in age, the ratios of MVPA:SB, LIPA:SB and TPA:SB steadily declined.

### Demographic and unadjusted adiposity characteristics across movemenet patterns

Table 2 reports crude demographic characteristics and adiposity markers among children and adolescents across the four movement patterns. Children in movement patterns 1 and 2 (considered more active individuals) were ≅ 2 years younger and had lower levels for each marker of adiposity when compared to children in movement pattern 4. For children, there were no statistical differences for race-ethnicity, PIR, cotinine, or energy intake across the 4 movement patterns. Among adolescents, those in movement pattern 1 were ≅ 1.5 years younger, had a lower cotinine level, and had a lower gynoid BF% and total BF% than those in movement pattern 4. Similar to children, there were no statistical differences for race-ethnicity or PIR across the 4 movement patterns for adolescents.

**Table 2:** Weighted unadjusted characteristics of children and adolescents who participated in NHANES 2003–2006 (mean/proportion [95 % CI])

	Movement Patterns; Mean/Proportion (95 % CI)			
	1	2	3	4
Variable	≥60 min/day MVPA and LIPA:SB ≥ 1	≥60 min/day MVPA and LIPA:SB < 1	<60 min/day MVPA but LIPA:SB ≥ 1	<60 min/day MVPA and LIPA:SB < 1
	Children (6–11 years)			
Mean Age (yr)	7.9 (7.7–8.1)*	8.4 (8.0–8.7)*	9.8 (9.4–10.1)	9.9 (9.6–10.1)
Gender, % ^a^				
Male	55.1 (50.5–59.8)	64.4 (56.1–72.7)	30.1 (15.6–44.7)	43.9 (36.0–51.8)
Female	44.8 (40.1–49.4)	35.5 (27.2–43.8)	69.8 (55.2–84.3)	56.0 (48.1–63.9)
Race-Ethnicity, %				
Mexican American/Other Hispanic	14.9 (9.7–20.0)	20.1 (11.9–28.4)	26.7 (12.0–41.4)	18.2 (12.5–24.0)
Non-Hispanic White	64.0 (55.6–72.4)	48.9 (37.8–60.1)	56.7 (40.3–73.0)	62.3 (51.5–73.2)
Non-Hispanic Black	13.8 (8.8–18.9)	17.0 (10.1–23.8)	11.1 (6.0–16.3)	11.7 (7.0–16.3)
Other Race	7.1 (3.3–10.9)	13.8 (5.7–21.9)	5.3 (0.0–11.4)	7.6 (1.3–13.8)
Poverty-to-Income Ratio	2.5 (2.2–2.8)	2.5 (2.1–2.9)	2.4 (1.9–2.9)	2.5 (2.1–2.9)
Cotinine (ng/mL)	0.53 (0.28–0.77)	0.48 (0.26–0.70)	0.69 (0.21–1.16)	0.28 (0.17–0.40)
Energy (kcal)	1978.7 (1928.6–2028.9)	2052.8 (1930.4–2175.2)	1973.3 (1802.2–2144.5)	1985.8 (1850.2–2121.4)
BMI (kg/m^2^)	17.2 (16.8–17.6)*	17.9 (17.1–18.6)*	20.3 (19.1–21.4)	20.8 (20.0–21.7)
BMI Percentile	61.3 (57.5–65.1)*	63.7 (57.4–70.0)*	71.9 (64.6–79.2)	72.5 (66.9–78.2)
Waist Circumference (cm)	61.3 (60.0–62.5)*	63.6 (61.4–65.7)*	70.2 (67.4–72.9)	73.6 (71.0–76.2)
Tricep Skinfold (mm)	11.3 (10.8–11.9)*	12.1 (11.0–13.2)*	15.7 (14.1–17.3)	17.3 (16.0–18.6)
Subscapularis Skinfold (mm)	7.9 (7.3–8.5)*	8.8 (7.7–9.8)*	11.2 (9.5–12.9)	12.1 (10.7–13.5)
Android Body Fat, %	**24.2 (22.5–26.0)**	**25.6 (23.3–27.9)**	31.5 (27.9–35.0)	32.4 (3.3–34.6)
Gynoid Body Fat, %	**32.3 (31.1–33.4)**	**32.6 (31.2–33.9)**	36.6 (34.6–38.5)	37.3 (36.0–38.7)
Total Body Fat, % ^b^	27.84 (0.56)*	29.1 (0.74)*	32.61 (1.03)	33.41 (0.66)
	Adolescents (12–17 years)			
Mean Age (yr)	**13.0 (12.7–13.4)**	**13.5 (13.1–13.9)**	**14.2 (13.9–14.5)**	14.6 (14.4–14.7)
Gender, % ^a^				
Male	89.1 (78.8–99.3)	86.0 (76.2–95.8)	60.6 (50.7–70.4)	44.3 (41.3–47.2)
Female	10.8 (0.6–21.1)	13.9 (4.1–23.7)	39.3 (29.5–49.2)	55.6 (52.7–58.6)
Race-Ethnicity, %				
Mexican American/Other Hispanic	17.6 (9.8–25.3)	27.4 (13.3–41.4)	10.7 (5.3–16.1)	16.9 (13.2–20.6)
Non-Hispanic White	59.3 (45.5–73.0)	47.7 (31.5–63.9)	72.1 (63.4–80.9)	63.5 (57.6–69.3)
Non-Hispanic Black	18.6 (10.3–26.8)	23.2 (14.2–32.1)	13.0 (8.5–17.5)	13.4 (10.4–16.5)
Other Race	4.4 (0.0–10.8)	1.6 (0.0–3.4)	4.0 (0.2–7.8)	6.0 (3.8–8.3)
Poverty-to-Income Ratio	2.5 (1.9–3.0)	2.6 (2.1–3.2)	2.6 (2.3–2.9)	2.8 (2.6–3.0)
Cotinine (ng/mL)	3.0 (0.0–6.3)*	9.8 (0.0–21.0)	7.3 (0.0–14.8)	9.7 (6.3–13.2)
Energy (kcal)	2514.9 (2178.3–2851.5)	2576.6 (2269.2–2884.0)*	2376.3 (2247.1–2505.6)*	2166.6 (2089.3–2243.9)
BMI (kg/m^2^)	21.5 (19.5–23.5)	21.9 (20.6–23.3)	22.5 (21.5–23.4)	22.7 (22.3–23.2)
BMI Percentile	63.2 (52.0–74.4)	66.5 (60.0–73.0)	65.7 (60.6–70.8)	65.8 (63.1–68.5)
Waist Circumference (cm)	76.0 (70.2–81.9)	77.7 (74.1–81.3)	79.1 (76.9–81.4)	79.4 (78.3–80.5)
Tricep Skinfold (mm)	14.0 (10.9–17.1)	14.8 (12.8–16.8)	15.3 (14.2–16.5)	16.0 (15.4–16.7)
Subscapularis Skinfold (mm)	11.2 (8.3–14.0)	11.9 (9.7–14.2)	12.3 (11.2–13.4)	13.2 (12.5–13.9)
Android Body Fat, %	23.2 (19.8–26.6)	24.9 (22.0–27.9)	25.9 (23.7–28.0)	27.6 (26.3–28.9)
Gynoid Body Fat, %	**27.6 (25.4–29.8)**	**28.2 (26.1–30.3)**	30.3 (28.3–32.2)	32.2 (31.2–33.1)
Total Body Fat, % ^b^	26.1 (1.3)*	25.8 (1.0)*	27.5 (0.8)	29.1 (0.3)

^a^ To make comparisons across lifestyle groups, linear regression was used for continuous variables. The referent group was the least active group (<60 min/day of MVPA and LIPA:SED < 1). To make comparisons across categorical variables, design-based likelihood ratio analyses was used. Asterik (*) (and bolded text) indicates Bonferroni-corrected statistical signifiance (*p* < 0.006)

^b^ For body fat percent, the mean estimate is the mean of the 5 multiple imputated estimates. The variance estimates are the mean of the 5 multiple imputated variance estimates. To examine body fat differences between groups, regression analysis was performed. Five separate regression models (one for each multiple imputation) were computed for each group. The square root of the total variance estimate ([within-imputation variance] + (6/5)*[between-imputation variance]) was calculated. The average regression coefficient was then divided by the square root of the total variance estimate to yield a *t*-value to determine if the mean estimate differed from the referent group. See statistical analysis section for more details

*SB* sedentary behavior, *LIPA* light-intensity physical activity, *MVPA* moderate-to-vigorous physical activity, *BMI* body mass index

### Adjusted adiposity characteristics across movement patterns

Table 3 shows the weighted multivariable linear regression results examining the association between markers of adiposity (outcome variable) and the movement patterns. Children in movement pattern 1, compared to those in movement pattern 4, had significantly lower levels of BMI (∆ 2.2 kg/m^2^), waist circumference (∆ 6.5 cm), tricep skinfold (∆ 4.2 mm), subscapularis skinfold (∆ 2.6 mm), android BF% (∆ 7.6 %), gynoid BF% (∆ 5.1 %), and total BF% (∆ 5.2 %). No results were significant for adolescents.

**Table 3:** Weighted multivariable linear regression associations between adiposity (outcome variable) and movement patterns and age groups

	Movement Patterns; Regression Coefficient (95 % CI) ^a^			
	1	2	3	4
Variable	≥60 min/day MVPA and LIPA:SB ≥ 1	≥60 min/day MVPA and LIPA:SB < 1	<60 min/day MVPA but LIPA:SB ≥ 1	<60 min/day MVPA and LIPA:SB < 1
Indice of Adiposity	Children (6–11 years)			
BMI (kg/m^2^)	**−2.2 (−3.3– −1.2)**	**−1.8 (−2.8– −0.8)**	−0.7 (−2.1–0.7)	Referent
BMI Percentile	−9.3 (−17.8– −0.7)	−7.2 (−15.6–1.0)	−2.0 (−11.5–7.5)	Referent
Waist Circumference (cm)	**−6.5 (−9.4– −3.5)**	**−5.3 (−8.2– −2.3)**	−3.7 (−7.6–0.1)	Referent
Tricep Skinfold (mm)	**−4.2 (−5.9– −2.5)**	**−3.6 (−5.5– −1.7)**	−2.0 (−4.0–0.02)	Referent
Subscapularis Skinfold (mm)	**−2.6 (−4.3– −0.9)**	−1.8 (−3.5– −0.2)	−1.2 (−3.2–0.6)	Referent
Android Body Fat, %	**−7.6 (−10.5– −4.7)**	**−5.7 (−8.9– −2.4)**	−2.5 (−6.4–1.4)	Referent
Gynoid Body Fat, %	**−5.1 (−7.0– −3.2)**	**−4.2 (−6.2– −2.3)**	−1.8 (−4.0–0.31)	Referent
Total Body Fat, % ^b^	**−5.2 (0.96)**	**−3.3 (1.05)**	−1.8 (1.07)	Referent
	Adolescents (12–17 years)			
BMI (kg/m^2^)	−0.2 (−2.2–1.7)	0.02 (−1.2–1.2)	−0.01 (−0.8–0.8)	Referent
BMI Percentile	−3.7 (−15.3–7.7)	0.4 (−5.9–6.7)	−0.5 (−5.8–4.7)	Referent
Waist Circumference (cm)	−1.5 (−7.2–4.1)	−0.2 (−3.7–3.2)	0.1 (−2.1–2.2)	Referent
Tricep Skinfold (mm)	0.2 (−2.7–3.3)	1.1 (−0.8–3.1)	0.02 (−1.2–1.2)	Referent
Subscapularis Skinfold (mm)	−0.2 (−3.3–2.7)	0.4 (−1.6–2.4)	−0.4 (−1.4–0.5)	Referent
Android Body Fat, %	−1.1 (−4.7–2.4)	0.52 (−2.5–3.5)	−0.65 (−2.6–1.3)	Referent
Gynoid Body Fat, %	−0.65 (−3.4–2.1)	−0.07 (−2.3–2.1)	−0.44 (−2.3–1.4)	Referent
Total Body Fat, % ^b^	−0.41 (1.2)	0.02 (1.0)	−0.42 (0.8)	Referent

^a^A separate multivariable linear regression analysis was computed for each adiposity marker (outcome variable) and for each age group (children vs. adolescents). Each model was adjusted for age, gender, race-ethnicity, cotinine, poverty-to-income ratio, accelerometer wear time, and energy intake

^b^For body fat percent, the regression coefficient is the mean of the 5 multiple imputated regression coefficients. The variance estimates are standard errors, which is reported as the square root of the total variance estimate ([within-imputation variance] + (6/5)*[between-imputation variance]). See statistical analysis section for more details

Bold indicates Bonferroni-corrected statistical significance (*p* < 0.006) when compared to referent the group

*SB* sedentary behavior, *LIPA* light-intensity physical activity, *MVPA* moderate-to-vigorous physical activity, *BMI* body mass index

### Adjusted adiposity characteristics comparing TPA to SB

Table 4 reports the weighted multivariable linear regression results examining differences in markers of adiposity between those who engaged in more TPA than SB (i.e., the ratio of TPA:SB was > 1). Children who engaged in more TPA than SB had lower levels of BMI (∆ 1.2 kg/m^2^), waist circumference (∆ 4.1 cm), tricep skinfold (∆ 2.3 mm), android BF% (∆ 4.5 %), gynoid BF% (∆ 2.7 %) and total BF% (∆ 2.9 %). No findings were significant for adolescents.

**Table 4:** Weighted multivariable linear regression associations between adiposity, physical activity and sedentary behavior

	Total Physical Activity ≥ Sedentary Behavior Regression Coefficient (95 % CI)^a^	
Variable	Yes	No
Indice of Adiposity	Children (6–11 years)	
BMI (kg/m^2^)	**−1.2 (−2.0– −0.3)**	Referent
BMI Percentile	−4.7 (−11.1– 1.6)	Referent
Waist Circumference (cm)	**−4.1 (−6.4– −2.0)**	Referent
Tricep Skinfold (mm)	**−2.3 (−3.7– −0.9)**	Referent
Subscapularis Skinfold (mm)	−1.7 (−2.9– −0.4)	Referent
Android Body Fat, %	**−4.5 (−7.1– −1.9)**	Referent
Gynoid Body Fat, %	**−2.7 (−4.5– −1.0)**	Referent
Total Body Fat, % ^b^	**−2.9 (0.9)**	Referent
	Adolescents (12–17 years)	
BMI (kg/m^2^)	−0.2 (−0.9–0.4)	Referent
BMI Percentile	−1.5 (−6.6–3.6)	Referent
Waist Circumference (cm)	−0.8 (−2.7–1.0)	Referent
Tricep Skinfold (mm)	−0.3 (−1.4–0.7)	Referent
Subscapularis Skinfold (mm)	−0.5 (−1.6–0.4)	Referent
Android Body Fat, %	−0.9 (−2.5–0.7)	Referent
Gynoid Body Fat, %	−0.6 (−2.0–0.7)	Referent
Total Body Fat, % ^b^	−0.6 (0.7)	Referent

^a^A separate multivariable linear regression analysis was computed for each anthropometric marker (outcome variable) and for each age group (children vs. adolescents). Each model was adjusted for age, gender, race-ethnicity, cotinine, poverty-to-income ratio, accelerometer wear time, and energy intake

^b^For body fat percent, the regression coefficient is the mean of the 5 multiple imputated regression coefficients. The variance estimates are standard errors, which is reported as the square root of the total variance estimate ([within-imputation variance] + (6/5)*[between-imputation variance]). See statistical analysis section for more details

Bold indicates Bonferroni-corrected statistical significance (*p* < 0.006) when compared to the referent group

### Adjusted adiposity characteristics across isotemporal substitution models

Table 5 shows the single behavior, partition, and isotemporal substitution models for each behavior with each marker of adiposity among children; results are only reported for children as associations were not significant for adolescents. Results were similar across the different markers of adiposity. For example, after applying adjustments (including age), the single behavior models (models 1–3) showed that SB and MVPA were associated with most markers of adiposity, with the association occuring in the expected direction; LIPA was not significant for any of the single behavior models. For all markers of adiposity, only MVPA was significant in the partition models.

**Table 5:** Single behavior, partition, and isotemporal substitution models for markers of adiposity among children (6–11 years)

	Regression Coefficient (95 % CI)^a^		
Model	SB	LIPA	MVPA
BMI			
Single Behavior (models 1–3)	**0.51 (0.23–0.78)**	−0.30 (−0.66**–**0.04)	**−1.24 (−1.66– −0.82)**
Partition (model 4)	0.15 (−0.06**–**0.37)	−0.02 (−0.38**–**0.32)	**−1.04 (−1.52– −0.56)**
Isotemporal substitution			
Replace SB (model 5)	Dropped	−0.18 (−0.53**–**0.16)	**−1.20 (−1.60– −0.79)**
Replace LIPA (model 6)	0.18 (−0.16**–**0.53)	Dropped	**−1.01 (−1.57– −0.45)**
Replace MVPA (model 7)	**1.20 (0.79–1.60)**	**1.01 (0.45–1.57)**	Dropped
BMI Percentile			
Single Behavior (models 1–3)	2.22 (−0.43**–**4.88)	−0.56 (−3.54**–**2.40)	**−7.29 (−11.96– −2.62)**
Partition (model 4)	0.48 (−1.37**–**2.34)	0.66 (−1.93**–**3.25)	**−6.84 (−11.56– −2.12)**
Isotemporal substitution			
Replace SB (model 5)	Dropped	0.17 (−2.60**–**2.95)	**−7.33 (−11.88– −2.78)**
Replace LIPA (model 6)	−0.17 (−2.95**–**2.60)	Dropped	**−7.50 (−12.53– −2.47)**
Replace MVPA (model 7)	**7.33 (2.78–11.88)**	**7.50 (2.47–12.53)**	Dropped
Waist Circumference			
Single Behavior (models 1–3)	**1.59 (0.83–2.36)**	−0.94 (−1.89**–**0.01)	**−3.95 (−5.26– −2.64)**
Partition (model 4)	0.27 (−0.31**–**0.87)	−0.27 (−1.17**–**0.62)	**−3.53 (−5.05– −2.01)**
Isotemporal substitution			
Replace SB (model 5)	Dropped	−0.55 (−1.50**–**0.38)	**−3.81 (−5.09– −2.54)**
Replace LIPA (model 6)	0.55 (−0.38**–**1.50)	Dropped	**−3.25 (−4.91– −1.59)**
Replace MVPA (model 7)	**3.81 (2.54–5.09)**	**3.25 (1.59–4.91)**	Dropped
Tricep Skinfold			
Single Behavior (models 1–3)	**0.90 (0.39–1.41)**	−0.40 (−1.01**–**0.20)	**−2.57 (−3.37– −1.78)**
Partition (model 4)	0.13 (−0.18**–**0.44)	−0.006 (−0.47**–**0.45)	**−2.41 (−3.17– −1.64)**
Isotemporal substitution			
Replace SB (model 5)	Dropped	−0.13 (−0.70**–**0.43)	**−2.54 (−3.30– −1.78)**
Replace LIPA (model 6)	0.13 (−0.43**–**0.70)	Dropped	**−2.40 (−3.31– −1.49)**
Replace MVPA (model 7)	**2.54 (1.78–3.30)**	**2.40 (1.49–3.31)**	Dropped
Subscapularis Skinfold			
Single Behavior (models 1–3)	**0.77 (0.32–1.22)**	−0.52 (−0.99**–** −0.05)	**−1.74 (−2.52– −0.97)**
Partition (model 4)	0.17 (−0.09**–**0.43)	−0.18 (−0.54**–**0.16)	**−1.48 (−2.22– −0.75)**
Isotemporal substitution			
Replace SB (model 5)	Dropped	−0.36 (−0.80**–**0.08)	**−1.66 (−2.37– −0.95)**
Replace LIPA (model 6)	0.36 (−0.08**–**0.80)	Dropped	**−1.30 (−1.97– −0.63)**
Replace MVPA (model 7)	**1.66 (0.95–2.37)**	**1.30 (0.63–1.97)**	Dropped
Android Body Fat Percent			
Single Behavior (models 1–3)	**2.06 (1.14–2.98)**	−1.20 (−2.41– −0.002)	**−6.75 (−8.71– −4.79)**
Partition (model 4)	0.17 (−0.38–0.73)	−0.10 (−1.23–1.01)	**−6.44 (−8.84– −4.05)**
Isotemporal substitution			
Replace SB (model 5)	Dropped	−0.28 (−1.59–1.02)	**−6.62 (−8.80– −4.45)**
Replace LIPA (model 6)	0.28 (−1.02–1.59)	Dropped	**−6.34 (−9.40– −3.27)**
Replace MVPA (model 7)	**6.62 (4.45–8.80)**	**6.34 (3.27–9.40)**	Dropped
Gynoid Body Fat Percent			
Single Behavior (models 1–3)	**1.29 (0.66–1.92)**	−0.69 (−1.46–0.07)	**−4.44 (−5.70– −3.18)**
Partition (model 4)	0.08 (−0.28–0.46)	0.01 (−0.69–0.71)	**−4.31 (−5.77– −2.86)**
Isotemporal substitution			
Replace SB (model 5)	Dropped	−0.07 (−0.87–0.71)	**−4.40 (−5.74– −3.07)**
Replace LIPA (model 6)	0.07 (−0.71–0.87)	Dropped	**−4.32 (−6.11– −2.54)**
Replace MVPA (model 7)	**4.40 (3.07–5.74)**	**4.32 (2.54–6.11)**	Dropped
Total Body Fat Percent ^b^			
Single Behavior (models 1–3)	1.47 (0.52)	−0.88 (0.57)	**−2.85 (0.75)**
Partition (model 4)	0.18 (0.42)	−0.08 (0.56)	**−4.42 (0.8)**
Isotemporal substitution			
Replace SB (model 5)	Dropped	−0.27 (0.58)	**−4.62 (0.77)**
Replace LIPA (model 6)	0.27 (0.58)	Dropped	**−4.34 (0.89)**
Replace MVPA (model 7)	**4.62 (0.77)**	**4.34 (0.89)**	Dropped

^a^Prior to the regression models, all physical activity variables were divided by a constant of 60 so that a unit increase in the behavior represented an increase of 60 min/day within the given behavior

^b^For body fat percent, the regression coefficient is the mean of the 5 multiple imputated regression coefficients. The variance estimates are standard errors, which is reported as the square root of the total variance estimate ([within-imputation variance] + (6/5)*[between-imputation variance]). See statistical analysis section for more details

Bold indicates Bonferroni-corrected statistical significance (*p* < 0.006)

*SB* sedentary behavior, *LIPA* light-intensity physical activity, *MVPA* moderate-to-vigorous physical activity, *BMI* body mass index

Covariates for models included age, gender, race-ethnicity, cotinine, poverty-to-income ratio, accelerometer wear time, and energy intake. Note, accelerometer wear time was not included in the partition or istemporal models (models 4–7) because accelerometer wear time is equal to the sum of each of the intensity categories

For model 1, SB and covariates were entered into the model

For model 2, LIPA and covariates were entered into the model

For model 3, MVPA and covariates were entered into the model

For model 4, SB, LIPA, MVPA and covariates were entered into the model to examine the unique effects of each intensity on the biomarker

For models 5–7, a total activity time (TAT) variable (SB + LIPA + MVPA) was entered into the models

For model 5, TAT, LIPA, MVPA and covariates were entered into the model (SB dropped)

For model 6, TAT, SB, MVPA and covariates were entered into the model (LIPA dropped)

For model 7, TAT, SB, LIPA and covariates were entered into the model (MVPA dropped)

Models 5–7 were used to estimate the substitution effect of replacing one behavior for another behavior. The behavior of interest is dropped from the model, which depicts the effect of replacing one behavior for another behavior

Isotemporal substitution models were similar across the different markers of adiposity. For total BF%, and after adjustments, replacing 60 min/day of SB with MVPA resulted in a 4.6 % decreased estimate of total BF% (model 5). Replacing 60 min/day of LIPA with SB resulted in a non-significant 0.3 % decreased estimate of total BF%. Lastly, substituting MVPA with SB (or LIPA) resulted in an increased estimate of total BF%.

## Discussion

The purpose of this study was to systematically examine the association between behaviors and movement patterns defined by SB, LIPA, and MVPA (separately and combined) with markers of adiposity among a large U.S.-representative sample of children and adolescents. We accomplished this by applying various traditional and novel analytical techniques, including an evaluation of movement patterns and isotemporal substitution models.

The major findings for *children* include:

- An inverse association between age and movement patterns;
- Children in movement patterns 1 and 2 had lower levels of most markers of adiposity (and were approximately 2 years younger) compared to children classified in movement pattern 4;
- Children whose TPA exceeded their SB had lower levels of markers of adiposity;
- MVPA was inversely associated with age and all markers of adiposity among children, independent of SB and LIPA; and
- Mathematically replacing 60 min/day of SB with MVPA was associated with a 4.6 % decreased estimate of total BF% among children.

Taken together, these findings suggest that as children age they engage in less LIPA and MVPA. Further, although children whose TPA exceeded their SB had lower BF%, only MVPA was associated with BF% in the partition models. Therefore, future longitudinal research should examine whether engagement in LIPA alone is insufficient in reducing adiposity, but when coupled with sufficient MVPA, reductions in adiposity among children occur.

The major findings for *adolescents* include:

- The very low prevalence (<5 %) of adolescents engaging in movement pattern 1; and that
- There were no significant multivariable associations of SB, LIPA, or MVPA with any marker of adiposity among adolescents. In the case of this last finding, a lack of significance may be a result of low statistical power as few adolescents actually engaged in movement pattern 1.

### Age and movement patterns

Children (52 %) were more likely than adolescents (4.7 %) to engage in movement pattern 1. The fact that < 5 % of U.S. adolescents engaged in movement pattern 1 is similar to results reported for Belgian adolescents (1 % for females and 6 % for males) [9]. Such a finding is typically lamented as a behavioral deficit, however, it is possible that as humans develop there is a natural progression to lower LIPA:SB and MVPA:SB ratios. This assertion is supported by the cross-sectional results depicted in the Figure, which show a steady decline in both MVPA:SB and LIPA:SB ratios with each successive increase in age. The cross-sectional inverse association apparent between age and movement patterns is similar to that reported in longitudinal studies that depict an age-related decline in MVPA [29]. The reasons for these age-related changes in children are likely multidimensional. From a developmental perspective, age-related decline in movement behaviors may be a result of altered neurotransmission of the dopaminergic system (i.e., age-induced reduction of dopamine) [30, 31]. From a psychological perspective, reduced perceived behavioral control and self-efficacy have predicted declines in physical activity (i.e., sports participation, physical activity-related energy expenditure, and MVPA) as children age [32]. Although not conclusive, body dissatisfaction associated with changes in development and pubertal status may also help to explain the age-related decline in MVPA [33]. From a social perspective, age-related decline in MVPA may also be from social expectations and pressures specific to this age that may displace time available for MVPA (e.g., dating or getting a driver’s license) [34]. Further, there is evidence that young people experience a decrease in nonorganized sports participation as they progress through the transition from childhood to adolescence and beyond [35].

As reported elsewhere [29], researchers have mostly examined age-related changes in MVPA. Our analyses also showed age-related cross-sectional inverse associations with LIPA:SB and TPA:SB, suggesting that age-related decreases in MVPA may also be accompanied by decreases in LIPA and increases in SB. Classification by the four general movement patterns also support these findings. If a compensatory increase in LIPA was tied to a decrease in MVPA, then we would observe a higher proportion of children and adolescents engaging in relatively more LIPA than SB if they did not meet the MVPA guideline. This compensatory effect is plausible as it may act as a mechanism to homeostatically control total daily energy expenditure. However, our analyses did not support this as only a few children (10 %) and adolescents (17 %) engaged in more LIPA than SB when they did not also meet the MVPA guideline; the opposite finding was more likely the case. Overall, these findings, along with others [29], suggest that promoting both MVPA and LIPA may be important in achieving and maintaining energy balance and optimal BF% as children age and develop.

### Movement patterns and markers of adiposity

Children demonstrated an association between movement pattern 1 and a lower total BF% estimate when compared to children classified into any of the other movement patterns. Results were also similar for android BF% and gynoid BF%, which is an important finding as android BF% and gynoid BF% are predictive of worse health outcomes (e.g., insulin resistance) in youth [36]. U.S. children in movement pattern 1 had approximately 5 % lower total BF% than those in movement pattern 4. Also, those in movement pattern 1 had approximately 2 % lower total BF% than those in movement pattern 2. Further, children whose TPA exceeded their SB had approximately 3 % lower total BF% estimate.

These findings are shaped by the fact that children in movement pattern 1 were also younger than their counterparts in the other movement patterns. Given the clear age associations with all types of physical activity behaviors noted above, coupled with the fact that certain markers of adiposity (e.g., abdominal circumference) increase as children age [37], strategies to maintain MVPA, LIPA, or TPA (ie., any type of movement behavior as opposed to SB) or attenuate the decline in these behaviors as children age, is pivotal.

Promoting more TPA among children may be feasible as children, compared to adolescents, naturally engage in more breaks from SB [38], which would logically promote TPA. Consequently, for adolescents, specific strategies to reduce SB and increase TPA, or replace SB with TPA, are needed. One such strategy for adolescents may be to make modifications to school-based activities (e.g., integrating movement into curriculum) as adolescents tend to engage in longer SB bouts during school when compared to nonschool hours [39].

### Independent MVPA association and isotemporal substitution paradigm

Not surprisingly, there appears to be an additive effect of LIPA and MVPA associated with lower marker levels of adiposity among children (i.e., movement pattern 1 had the lowest BF% estimate). This supports the contention that both contribute to overall energy balance. In addition, there was evidence of an independent association of MVPA with markers of adiposity among children, which is consistent with other studies among children [7]. Our findings do not, however, demonstrate independent associations of LIPA or SB with markers of adiposity, which is consistent with the findings of a recent review [40] concluding that, among children and adolescents, the association between SB and health is attenuated after controlling for MVPA. Similarly, some [6], but not all [2] studies in this population have shown that the association between LIPA and adiposity and other health markers is attenuated after controlling for MVPA. Taken together, these cross-sectional findings *suggest* that LIPA alone may not be sufficient to improve health outcomes in children, but when coupled with sufficient MVPA, reductions in adiposity among children may occur.

Lastly, the isotemporal substitution model revealed that replacing 60 min/day of SB with MVPA is associated with a 4.6 % decreased total BF% estimate. These findings are congruent with the emerging work in the adult population showing that replacing LIPA with MVPA is associated with improved cardiovascular disease risk profile [13], and replacing MVPA with LIPA results in worse outcomes (e.g., increased weight gain) [12]. As a result, efforts among children are needed to help replace at least some SB with MVPA. Since television watching is a primary source of children’s leisure time sedentary behavior [41], one potential strategy may be to purchase devices that link the television’s power supply to children’s physical activity engagement [42]. Another potential strategy to accomplish this is to integrate high-intensity fitness bouts during children’s school-based recess [43]. For example, school-based fitness bout interventions have consisted of a 15-min bout of high-intensity physical activity where children engaged in a 400-m obstacle course that contained MVPA activities such as running and crawling.

### Strengths and limitations

Strengths of this study include using various traditional and novel analytical techniques to consider the combined and separate associations between SB, LIPA and MVPA with several markers of adiposity. Other strengths include using an objective measure of android, gynoid, and total BF%, assessing accelerometer-measured SB, LIPA, and MVPA, and employing a large U.S.-representative sample of children and adolescents. Despite these strengths, this study is not without limitations. For example, the cross-sectional design renders temporality, and thus causal conclusions, impossible. Another limitation is the inability to control for sleep (only NHANES participants ≥ 16 years were eligible for the sleep questionnaire), as sleep has been shown to associate with markers of adiposity among adolescents [44]. Sleeping patterns may also influence movement patterns [45]. Therefore, future research on this topic should statistically control for sleep. When feasible, studies should examine the potential effect that sleeping patterns may have on the relationship between movement patterns and adiposity. Lastly, the average accelerometer wear time in the present study for children and adolescents was approximately 14 h, suggesting that participants slept for 10 h, which is consistent with sleep duration estimates for children [46]. However, average sleep duration for adolescents appears to be around 8 h/night [47]. Therefore, future research, particularly among adolescents, should identify effective strategies to promote wearing the accelerometer during all waking hours. Alternatively, having participants wear the accelerometer for the entire 24-h day may help avoid monitoring non-compliance and its associated biases [48].

### Further direction for future research

The drastic differences in movement patterns and MVPA between U.S. children and adolescents raises the question of whether MVPA guidelines for adolescents should be refined to account for the developmental changes that occur during the transition from childhood to adolesecence. Currently, the MVPA guideline of 60 min/day is identical for children and adolescents [25], despite the noted developmental differences (e.g., puberty-induced anxiety and self-conscious [49], and age-induced reduction of neurotransmitters [30, 31]) between children and adolescents that may influence this age-related decline in MVPA. Further, others have critically evaluated the scientific evidence on which these guidelines are based and concluded that the evidence is weak [50] and requires rethinking of current recommendations [51]. Although recent comprehensive reviews [52] have supported the 60 min/day recommendation for adolescents after examining 850 studies, most of the intervention studies that informed this recommendation did not examine the efficacy of 60 min/day of MVPA, but rather demonstrated beneficial effects of 30–45 min/day of MVPA [51]. Clearly, additional research and critical thought is needed, but a sensible MVPA threshold to further consider for improving markers of health, including adiposity, may be 60, 45, and 30 min/day, respectively, for children, adolescents, and adults. Lastly, before such recommendations are developed, future research examining the longitudinal validity of the accelerometer-derived, age-specific activity count cut-points is needed. The age-specific activity count cut-points were derived from cross-sectional analyses, and to be fully certain of an age-related decline in accelerometer-determined physical activity, longitudinal validity of these activity count cut-points against some unit of energy expenditure (e.g., indirect calorimetry) is urgently needed.

With regard to the isotemporal substitution analysis, we demonstrated that substituting 60 min/day of SB with MVPA was associated with approximately 5 % reduced BF% estimate among children. Longitudinal data is needed to confirm these analyses, but this sheds some light on the important public health question of how children should spend some of their time for weight control purposes. However, for children and adolescents who have a differential preference with respect to physical activity intensity, it would be useful to determine if LIPA has a similar effect on BF% when compared to an equal amount of calories expended for MVPA. It may be assumed that if caloric intake is unchanged and the caloric expenditure for the two behaviors (LIPA and MVPA) are equal, this will have an identical effect on BF% change. However, this may not be the case as, for example, physical activity intensity may have a differential effect on post-exercise oxygen consumption [53], ultimately influencing total daily energy expenditure. Therefore, future longitudinal research should consider estimating the isocaloric substitution effect of energy expenditure on one behavior for energy spent on another behavior while ensuring caloric intake is unchanged. Such an isocaloric expenditure analysis may help individuals choose an appropriate physical activity intensity level based on their physical activity intensity preference.

In conclusion, this secondary data analysis of children’s and adolescents’ data showed a clear inverse relationship between age and movement patterns, with increased age associated with lower ratios of MVPA:SB, LIPA:SB and TPA:SB. Further, a large proportion of U.S. children (48 %) and U.S. adolescents (95 %) did not engage in ≥ 60 min/day of MVPA and accumulated relatively less LIPA than SB, with prevalence estimates similar across genders. The low proportion of children engaging in ≥ 60 min/day of MVPA and accumulating relatively more LIPA than SB had the lowest android BF%, gynoid BF%, and total BF%. Similarly, children whose daily TPA exceeded their SB had a lower android BF% (∆ 4 %), gynoid BF% (∆ 3 %), and total BF% estimate (∆ 3 %). Further, substituting 60 min/day of SB with MVPA was associated with a reduced android BF% (∆ 6 %), gynoid BF% (∆ 4 %) and total BF% estimate (∆ 5 %). Notably, these findings are likely driven by the participant’s age, as children in movement pattern 1 and children whose TPA exceeded their SB were the youngest. Future longitudinal and experimental research is needed to better tease out whether the association between movement patterns and adiposity is driven by a physical activity change or an age/developmental change.

Taken together, these findings coupled with the fact that certain markers of adiposity (e.g., abdominal circumference) increase as children age [37], underscore the importance of maintaining MVPA, LIPA, or TPA or attenuate the decline in these behaviors as children age. Lastly, given that MVPA was the only independent predictor in our cross-sectional partition model, future longitudinal research should examine whether LIPA alone is insufficient in reducing adiposity, but when coupled with sufficient MVPA, results in reductions in adiposity among children.
